# The Fetal Hypothalamus Has the Potential to Generate Cells with a Gonadotropin Releasing Hormone (GnRH) Phenotype

**DOI:** 10.1371/journal.pone.0004392

**Published:** 2009-02-06

**Authors:** Roberto Salvi, Yvan Arsenijevic, Marco Giacomini, Jean-Pierre Rey, Marie-Jeanne Voirol, Rolf Christian Gaillard, Pierre-Yves Risold, François Pralong

**Affiliations:** 1 Service of Endocrinology, Diabetology and Metabolism, University Hospital, Lausanne, Switzerland; 2 Jules Gonin Eye Hospital, University Hospital, Lausanne, Switzerland; 3 Service of Endocrinology, Diabetology and Metabolism, University Hospital, Geneva, Switzerland; 4 Laboratoire d'Histologie, EA 3922, Faculté de Médecine et de Pharmacie, Besançon, France; The University of Queensland, Australia

## Abstract

**Background:**

Neurospheres (NS) are colonies of neural stem and precursor cells capable of differentiating into the central nervous system (CNS) cell lineages upon appropriate culture conditions: neurons, and glial cells. NS were originally derived from the embryonic and adult mouse striatum subventricular zone. More recently, experimental evidence substantiated the isolation of NS from almost any region of the CNS, including the hypothalamus.

**Methodology/Findings:**

Here we report a protocol that enables to generate large quantities of NS from both fetal and adult rat hypothalami. We found that either FGF-2 or EGF were capable of inducing NS formation from fetal hypothalamic cultures, but that only FGF-2 is effective in the adult cultures. The hypothalamic-derived NS are capable of differentiating into neurons and glial cells and most notably, as demonstrated by immunocytochemical detection with a specific anti-GnRH antibody, the fetal cultures contain cells that exhibit a GnRH phenotype upon differentiation.

**Conclusions/Significance:**

This *in vitro* model should be useful to study the molecular mechanisms involved in GnRH neuronal differentiation.

## Introduction

NS were first discovered by Reynolds and Weiss [1], [2], as colonies of cells containing neural stem and precursor cells in embryonic and adult mouse striatum subventricular zone. These primary NS could be expanded in vitro by mechanical dissociation to single cells that generated new (secondary) NS, in a process that can be repeated many times. Furthermore, cells in each NS could be induced to differentiate into neurons and glial cells, thus demonstrating the two cardinal features of stem cells: self-renewal and multipotentiality. This initial report fueled many other investigations that further confirmed and substantiated the idea that neurospheres can be isolated from many other regions of the CNS of rodents and humans [3]–[7]. Overall, these results suggest that the adult CNS has strong neurogenic potential, presumably due to the presence of putative stem and progenitor cells. The precise nature of these cells is still a matter of investigation and controversy [8]–[10]. It is assumed that under normal conditions, these cells are kept in a quiescent state by inhibitory signals, but can be induced to proliferate upon exposure to adequate growth factors, most notably EGF and FGF-2 [11]–[13]. The recent finding by Markakis et al. [14] that GnRH-immunoreactive cells can be derived from in vitro expanded adult progenitor cells prompted us to set up a NS assay using either fetal or adult rat hypothalamic tissue and verify if we could detect cells with GnRH-phenotype in these cultures. The advantage of the NS assay is that stem/progenitor cells can be 1) isolated and propagated in a serum free medium, 2) studied at clonal density, and 3) stimulated to induce the differentiation of the whole population of the growing cells. This method should also help to determine whether fetal CNS progenitors have the potential to generate GnRH neurons. Here we report that both the fetal and the adult rat hypothalami are a rich source of NS that can be expanded and passaged for a long time, with the capability to give rise to neurons and glial cells under differentiating conditions. In addition, we report the detection of GnRH-immunoreactive cells among differentiating NS derived from fetal cultures.

## Results

### Hypothalamic tissue is a rich source of neurospheres with self-renewing capacity

**Figure 1:**
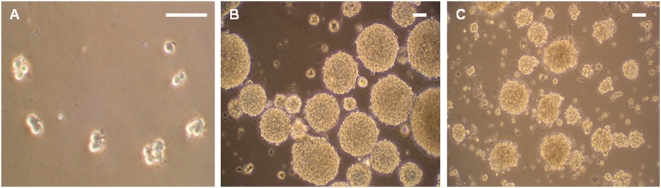
Morphology of hypothalamic-derived NS, phase-contrast images. A, Dividing cells after 2 days in vitro (2DIV). B, Fetal hypothalamic NS after 7DIV. C, Adult hypothalamic NS after 7DIV. Scale bars: 50 μm.

**Figure 2:**
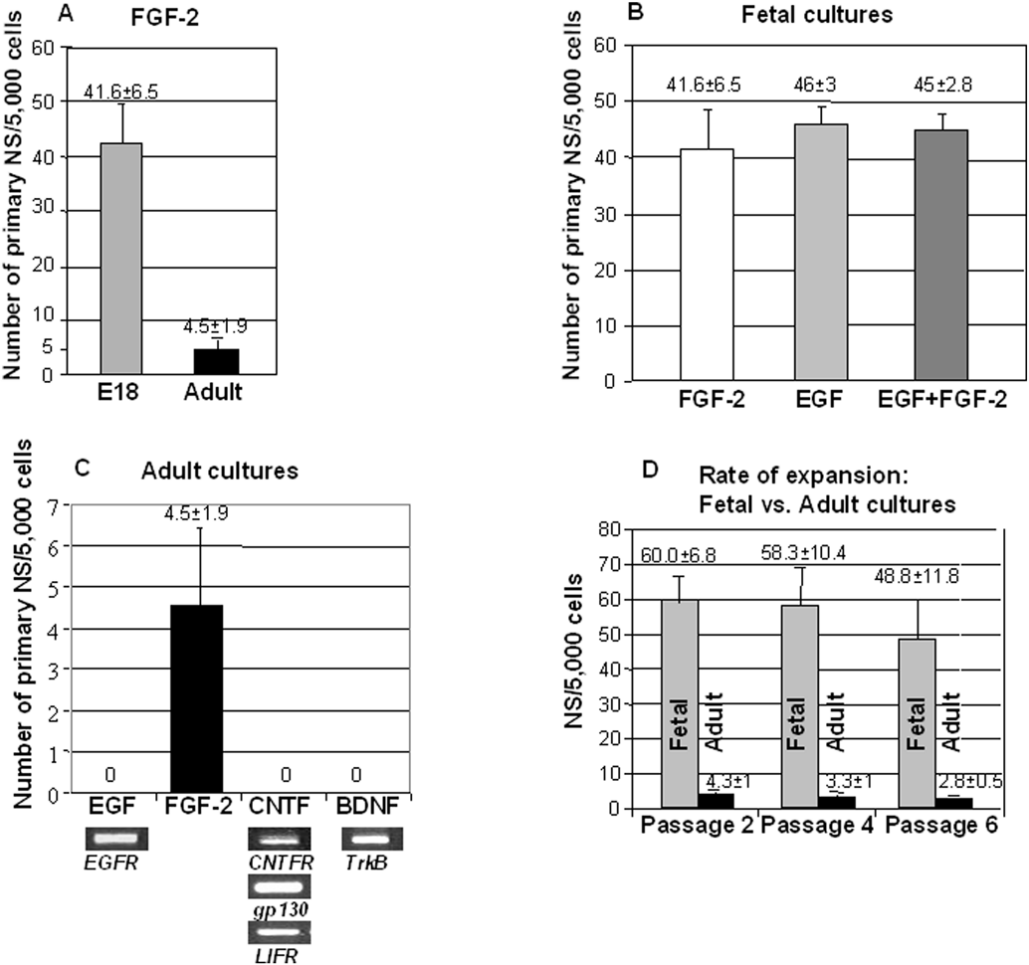
Efficiency of primary NS formation in the presence of different growth factors and rate of expansion of fetal vs. adult NS. A, Comparison of the number of NS formed in either fetal or adult hypothalamic cultures in the presence of FGF-2 after 7DIV. B, Comparison of the numbers of primary NS formed in fetal hypothalamic cultures in the presence of either EGF, FGF-2 or EGF plus FGF-2 after 7DIV. C, Comparison of the numbers of primary NS formed in adult hypothalamic cultures in the presence of either EGF, FGF-2 or EGF plus FGF-2 after 7DIV. The insets underneat this graph show the expression of the growth factor receptors for EGF (*EGFR*), CNTF (*CNTFR/gp130/LIFR* ) and BDNF (*TrkB*), as detected by RT-PCR in the RNA prepared from adult hypothalami. D, Comparison of fetal vs. adult NS rate of expansion upon serial passages, all cultures were kept in the presence of FGF-2 plus EGF and counted after 7DIV, respectively before passage 2 (secondary NS), 4 or 6. Values are average number of NS±SEM (n = 3 or 4 independent experiments).

Hypothalami were recovered from E18 embryos (Wistar rats) and after tissue dissociation, cells were seeded into 6-well plates at a density of 5,000 viable cells/mL, and cultured in the presence of FGF-2 (20 ng/mL), which was suggested to be necessary for neural stem cell renewal during early stage of the CNS development [15]–[17]. After a few days in culture, cell division was observed and small spherical cell colonies forming clusters begun to appear (Fig. 1A). At 7 days in vitro (7DIV), many clusters of different sizes ranging from small spheres to large colonies were formed (Fig. 1B). Using the same culture system, NS were also obtained from adult (8 to10 weeks old) rat hypotalami in the presence of FGF-2. These were morphologically similar to their embryonic counterparts (Fig. 1C), but the number of spheres generated from adult hypothalami was significantly lower and their size were smaller. A comparison of the different yields between fetal and adult cultures is shown in Fig. 2A. Next we tested the effect of EGF, the other growth factor commonly used to induce the formation of NS and which appeared to control later stages of neural stem cells [15]–[17]. In the E18 fetal cultures, EGF (20ng/mL) was as effective as FGF-2 after 7DIV (Fig. 2B). The use of both FGF-2 and EGF did not show any additive effect in these cultures (Fig. 2B), suggesting that in the fetal hypothalami there is probably only a single population of stem/progenitor cells which is expressing both receptors. In contrast, in adult cultures, no NS were obtained in the presence of EGF at the 7DIV time point (Fig. 2C), despite the fact that the receptor for EGF (EGFR) was expressed in the adult hypothalamic tissue prior to culture (Fig. 2C inset below graph). In fact, much longer incubation time was required (>3 weeks) to allow the formation of very few and small colonies (data not shown). Secondary and later passage NS derived from FGF-2-responsive primary NS expressed FGFR1, the receptor for FGF-2, but not EGFR (data not shown). CNTF and BDNF exhibit strong neurogenic properties in vivo in the hypothalamus of adult mice and rats [18], [19]. Therefore, we also tested the potential capacity of these two factors to support NS formation in our adult hypothalamic cultures. Despite the fact that the receptors for both molecules (respectively CNTFR/Gp130/LIFR and TrkB) were expressed in adult hypothalami prior to culture (inset below graph Fig. 2C), no NS formation was seen after 7DIV (Fig. 2C), nor after more extended time periods of incubation (until 3 weeks, data not shown). Overall, our results indicate that fetal and adult hypothalamic tissues are a rich source of cells capable of proliferation in vitro, upon stimulation with either FGF-2 or EGF, the latter being effective only in fetal cultures. In addition, fetal and adult primary NS could be passaged and expanded many times, by simple mechanical dissociation into single cells. A comparison of the rate of expansion of fetal vs. adult NS upon serial passaging is shown in Fig. 2D. These cultures were kept in the presence of FGF-2 plus EGF and NS counted after 7DIV, respectively before the 2^nd^ (secondary NS), 4^th^ and 6^th^ passage. Some adult cultures could be kept until the 12^th^ passage and stored as frozen cultures.

### NS can be generated from the hypothalamic parenchyma

**Figure 3:**
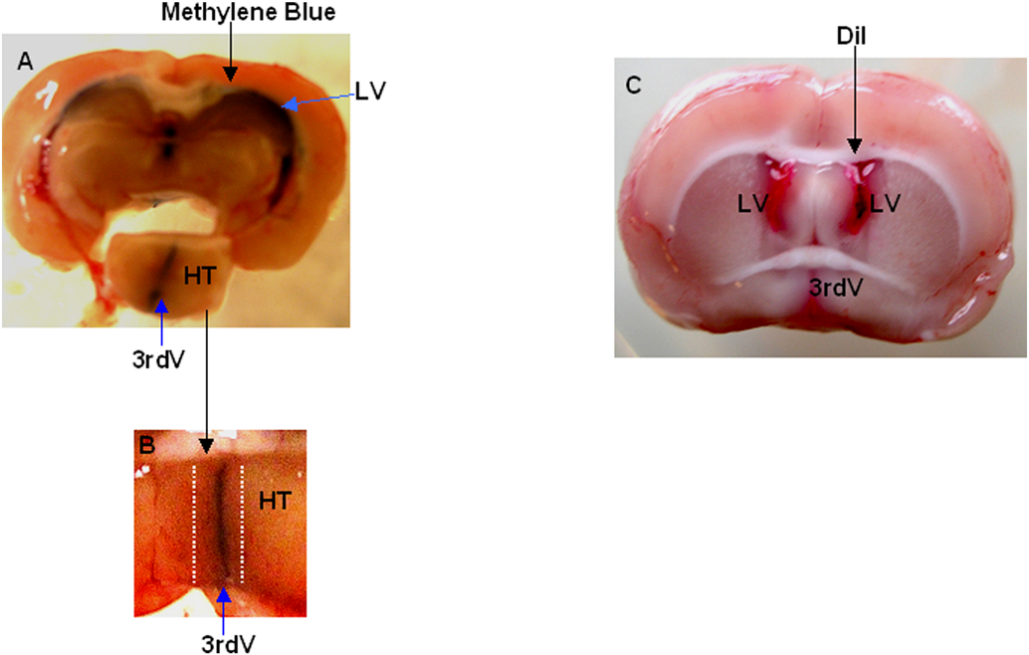
Representative images of brains sectioned after lateral ventricle administration with either methylene blue or DiI dyes. A, Coronal section of adult rat brain injected icv with methylene blue, showing the dissected hypothalamus (HT), (LV: lateral ventricle), (3rdV: third ventricle). B, Enlargement of HT showing the region (dotted lines) that was removed prior to starting cell culture. C, Coronal section of adult rat brain after icv DiI injection, laterals and third ventricles are red stained by the dye.

Since a recent report demonstrated the presence of neuronal precursor cells in the ependymal layer of the third ventricle in adult rats, resulting in the generation of NS in vitro [20], we next questioned whether NS obtained from adult rat cultures could be derived from precursors cells residing within the hypothalamic parenchyma. To this end, we completely removed the ependymal layer and some of the tissue immediately surrounding the third ventriculum, before starting the culture preparation. This was accomplished by administering a dye solution (either methylene blue or DiI) into the lateral ventricle of anesthetized adult rats, subsequently removing the brain and then dissecting out the entire hypothalamus. This is shown in Fig. 3A, in this case the dye methylene blue diffused and labels the third ventricle, making it easy to completely remove the blue stained third ventriculum, including some of the surrounding hypothalamic tissue (part underlined in Fig. 3B). DiI was also effective in staining (in red) the ventricular tissues (Fig. 3C). Therefore, the cultures derived from hypothalami lacking the third ventricle ependymal layer and surrounding tissue should be devoid of contaminating ependymal-derived precursor cells. Using this procedure, we were able to generate NS with the same efficiency as when the entire hypothalamus including third ventriculum was used (data not shown). In addition, these cultures had similar responses to growth factors as the previous cultures obtained from hypothalami containing third ventricle tissue: only FGF-2, but not EGF, was effective in stimulating NS formation. Immunofluorescence analysis of the NS obtained from DiI-injected animals showed that none of the cells within the NS incorporated the DiI fluorescent dye. In contrast, parallel cultures from ventricular tissue which had been stained with DiI failed to generate NS after a two-week incubation.

### Hypothalamic NS express Neuronal, Glial and Neuroepithelial markers, and can differentiate into Neuron,Astrocyte and Oligodendrocyte cells

**Figure 4:**
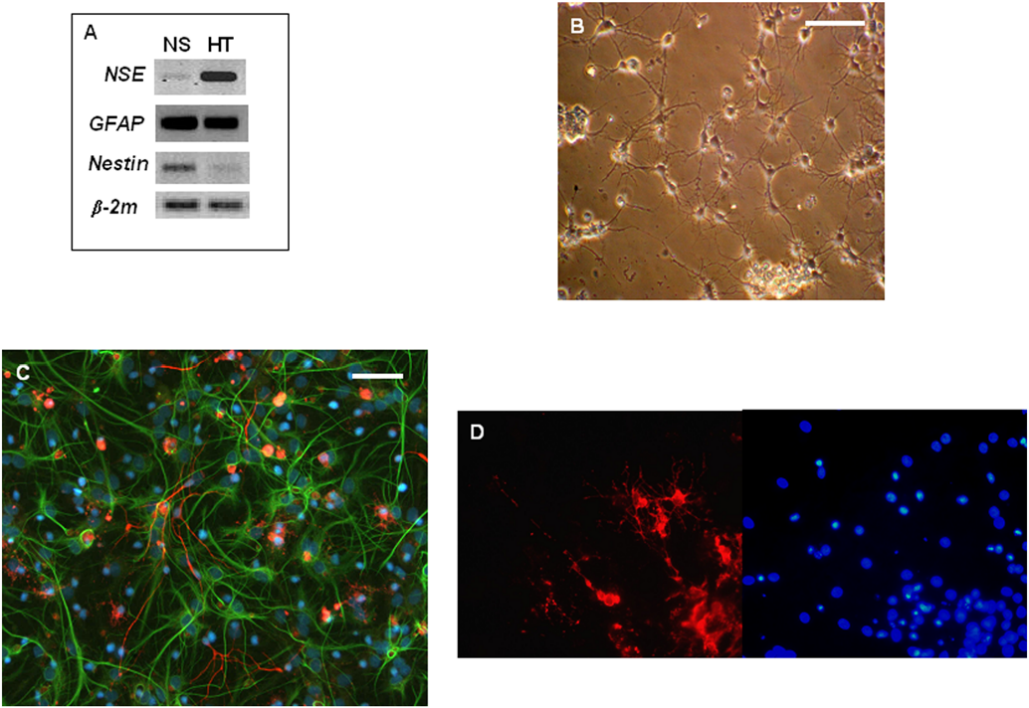
Hypothalamic NS cultures harbor precursors that can differentiate in neurons and glial cells. A: Gene expression analysis of some lineage-specific CNS markers by RT-PCR. RNA was isolated from hypothalamic-derived fetal secondary NS and adult hypothalami (HT) used for comparison: *NSE*, a marker for neurons, *GFAP*, a marker for astrocytes and *Nestin*, a marker for undifferentiated/neuroepithelial cells. β-2 microblobulin ( *β-2m*) was used as housekeeping gene. B: Phase-contrast image showing the typical morphology of differentiating NS cultures after 7DIV. C: Representative fluorescence micrograph after double-labeling immunofluorescent detection of neuronal and astrocyte cells in fetal differentiating cultures (7DIV) by using antibody lineage-specific markers: βTubIII (red: neurons) and GFAP (green: astrocytes). Nuclei are stained in blue by the dye DAPI. D, fluorescence micrograph after immunofluorescent detection of oligodendrocyte cells (showned here in red) in fetal differentiating cultures (7DIV) by using an antibody oligodendrocyte-specific marker: O4 (left side). DAPI-stained nuclei from same micrograph shown on right side. Scale bars: 50 μm.

To characterize the hypothalamic NS obtained and establish whether cells forming the NS exhibit multipotentiality, the expression of a panel of cell-type specific marker genes such as NSE (neuron specific enolase neuronal marker), GFAP (astrocyte marker) and Nestin (neuroepithelial/undifferentiated cell marker) was studied by RT-PCR. The NS generated within each separate culture were pooled and processed in order to prepare RNA which was then used for RT-PCR analysis. For these experiments, we only used NS derived from passaged primary fetal and adult NS (secondary and tertiary NS), to avoid any possibility of contamination with cells derived from primary culture. Fig. 4A shows the results of a representative experiment from adult NS (n = 4 samples from independent cultures analyzed), confirming the expression of all the cell markers assessed. These are compared to the expression of the same markers in RNA extracted from adult rat hypothalami (HT). Beta-2 microglobulin gene (β-2m) was chosen as housekeeping gene to verify sample quality and PCR conditions. NS isolated from other parts of the CNS could differentiate into neurons and glial cells when cultured under appropriate differentiating conditions, usually in media supplemented with 1–2% FBS but without the presence of the mitogens EGF/FGF-2. Therefore, we next verified whether the hypothalamic-derived NS could differentiate into both neurons and glial cells. Either secondary or tertiary NS were mechanically dissociated, and the resulting single-cells were cultured in a media devoid of growth factors but supplemented with 1% FBS. Cells kept under this condition attached to the surface of vessels and started to assume differentiated morphologies (see Fig. 4B). A representative picture from immunocytochemical analysis of fetal cultures at 7DIV is displayed in Figure 4C and demonstrates that the major CNS phenotypes can be detected, using specific antibodies for neurons (beta Tubulin III, red staining), and astrocytes (GFAP, green staining). Cells immunoreactive for the neuronal marker beta Tubulin III represented 5±1.5% of the total population derived from fetal NS (n = 3 cultures analyzed, from independent differentiating secondary and tertiary NS cultures). Parallel analysis for differentiated NS derived from adult NS gave similar results (data not shown). In addition, we also were able to detect, in fetal differentiating cultures, cells expressing the oligodendrocyte marker O4 (see Fig. 4D).

### Hypothalamic-specific transcription factors are expressed in NS derived from adult and fetal rat hypothalami

**Figure 5:**
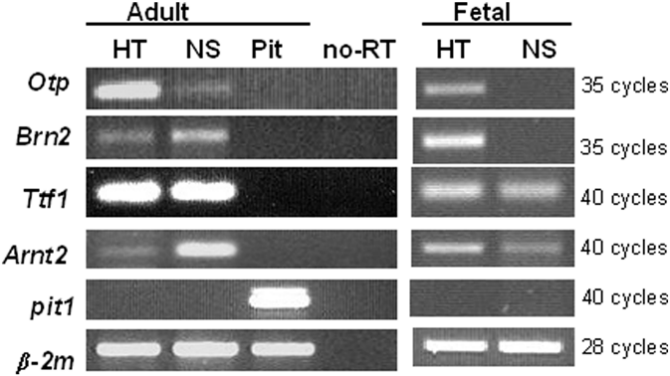
Hypothalamic-derived adult and fetal NS express some region-specific transcription factors. Gene expression analysis was determined by RT-PCR. RNA was isolated from adult and fetal tertiary NS cultures, adult and fetal hypothalami (HT) and adult anterior pituitary glands (Pit). Primers were designed to detect *Otp* (454 bp), *Brn2* (390 bp), *Ttf1* (128 bp), *Arnt2* (162 bp), *Pit1* (505 bp), and the housekeeping gene *β-2m* (142bp) used as control. The number of cycles performed for the PCR reactiones were indicated for each gene on the right side.

The transcription factors Otp, Brn2, Arnt2 and Ttf1 have been shown to play a pivotal role in the development of different neuroendocrine cell lineages within the hypothalamus [21]–[23]. To assess whether hypothalamic-derived NS retained characteristics related to the region from which they were isolated, the expression of these transcription factors was assessed by RT-PCR in adult and fetal secondary/tertiary NS. As shown in Fig. 5, these factors were all expressed in both adult and fetal hypothalamic RNA extracts, as expected, as well as in RNA extracts from the adult NS cultures. In contrast, in RNA extracts from fetal NS only Ttf1 and Arnt2 were detected (right side of Fig. 5). Of note, none of these factors was expressed in the adult anterior pituitary gland, used as a control, which instead expressed the pituitary-specific transcription factor Pit1 [24]. Pit1 was neither detected in the adult hypothalamus nor in the adult/fetal NS. Overall, these results indicate that our hypothalamic-derived NS cultures retain some specificities typical of the region from which they are derived.

### GnRH-Immunoreactive cells are present among differentiating NS of Fetal origin

**Figure 6:**
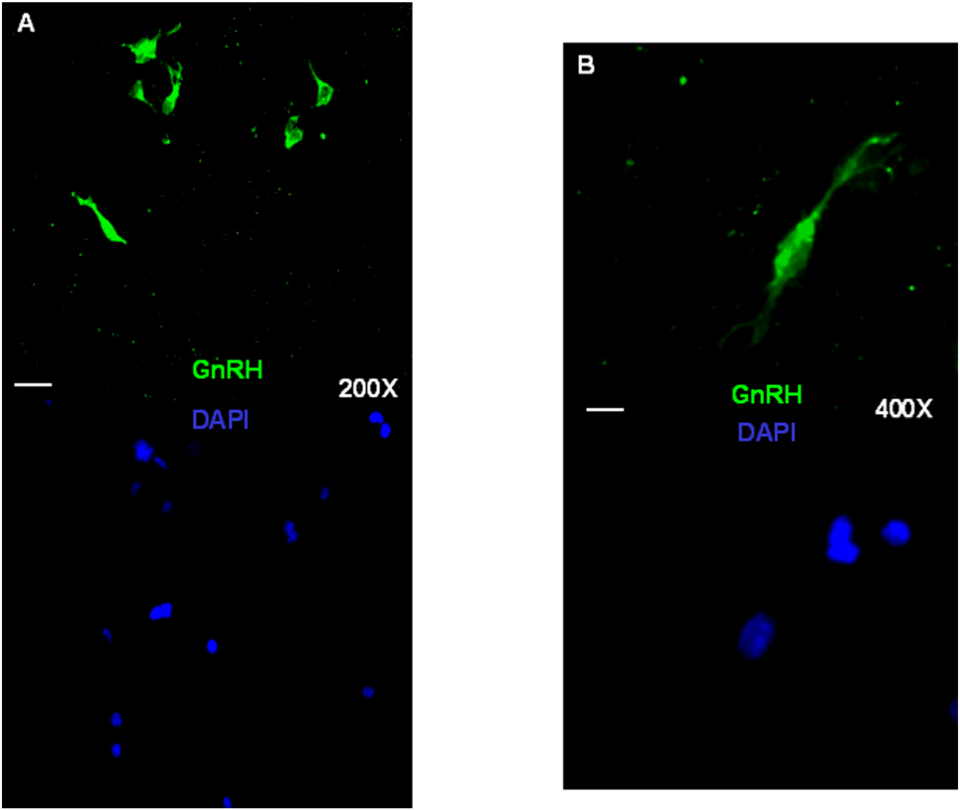
GnRH-immunoreactive cells as detected by a specific anti-GnRH antibody in differentiating fetal hypothalamic NS cultures. Representative fluorescence micrographs showing some GnRH-labeled immunofluorescent cells (green fluorescence) as detected in differentiating cultures kept for 5 days in DMEM/F12, FGF-2 (20ng/mL) followed by 5 days in DMEM/F12, B27 (0.5%). Duplicate pictures show the nuclei stained in blue by the dye DAPI. Scale bars: 20 μm (A panel) and 10 μm (B panel).

We next wondered if cells with a GnRH phenotype could be present in these hypothalamic-derived NS cultures after differentiation. To detect GnRH expressing cells, we performed immunocytochemistry on cells fixed in 4% paraformaldeyde using either a polyclonal antibody directed against GnRH [25] or a commercially available anti-GnRH antibody. Differentiated cultures were first studied after 7DIV in standard NS medium containing 1% FBS but without growth factors, and on poly-D-Ornithine treated coverslips. GnRH immunoreactive cells were indeed detected at the end of this incubation and represented an average of 10±0.5% of all cells (n = 3 cultures analyzed, from independent differentiating tertiary NS cultures). We next tried several other incubation media and finally were able to enrich the GnRH-immunoreactivecells population by using a differentiating protocol which consisted in incubating dissociated tertiary fetal NS for 5 days in a DMEM/F12 plus FGF-2 (20ng/mL) media followed by a further 5 days incubation in a DMEM/F12 plus B27 (0.5%) media(cells grown on poly-D-Ornithine treated coverslips). Under these conditions, the GnRH-immunoreactive cells (Fig. 6, green fluorescent cells) represented 21±2% of all cells analyzed (n = 3 cultures analyzed, from independent differentiating tertiary NS cultures), thus basically doubling the yeald obtained from the previous protocol.In contrast, no GnRH-immunoreactive cells could be detected in adult derived NS (data not shown)

## Discussion

We report here that primary cultures obtained from either fetal (E18) or adult rat hypothalamic tissue are an abundant source of neuronal precursor cells that proliferate in culture as free-floating spheres. By supplementing standard hypothalamic primary cultures [26] with growth factors such as EGF and FGF-2, we were able to reproducibly obtain large amounts of NS that can be maintained for a long period of time while retaining the capability of differentiating into the principal CNS cell phenotypes: neurons and glial cells. These data are in agreement with a recent report demonstrating the generation of NS from explants derived from small, anatomically-defined, subregions of adult rat CNS, including parts of the hypothalamus [6]. Interestingly, these authors also found that hypothalamic explants were among the richest sources of NS compared with other regions of the CNS such as the hippocampus.

We found here that the different growth factors used in the cultures influenced differently the development of NS in fetal compared to adult hypothalami. In fetal cultures, EGF and FGF-2 were almost equally efficient in promoting the generation of primary NS, but without exhibiting any additive effect. This observation implies that the same population of progenitor/stem cells is responding to stimulation with either growth factor, suggesting the expression of their two specific receptors in the same cells. In cultures from adult hypothalami, generation of NS was obtained effectively and reliably only with FGF-2, despite our concomitant finding that the EGFR is expressed in adult hypothalami. This observation is nevertheless consistent with previous data [20], but its significance remains to be elucidated.

We were also able to demonstrate that these NS are derived from the hypothalamic parenchyma, at least in the adult rat cultures, since the entire third ventricle lining including its immediately surrounding tissue was removed before culturing. This point appears important because the ependymal and subependymal layers of the lateral ventricles were once believed to be the only regions where putative neural progenitors/neural stem cells reside [27]. Consistently, more recent data show that the ependymal layer of the third ventricle also contains neuronal precursor cells capable of proliferation and migration within the hypothalamic parenchyma [20]. However, it is now relatively well established that parenchymal tissue from different CNS regions such as the cortex, septum, striatum and the optic nerve contain quiescent neuronal progenitors that can be induced to proliferate in vitro [4], [7], [11]. In our system, the hypothalamic-derived NS cultures were able to retain some phenotypic specificity of their area of origin. We could notably demonstrate the expression of transcription factors essential for the differentiation of many neuroendocrine cell lineages of the hypothalamus [21]–[23] in NS cultures from adult and fetal hypothalami. This result is entirely in line with the demonstration that neural stem cells and their progeny are regionally specified in the developing brain [28].

The postnatal hypothalamus has long been considered a neurogenically silent region with no proliferative capacity, but recent *in vivo* studies are challenging this hypothesis: in rats, the icv infusion of BDNF results in the accumulation of BrdU-positive cells in several CNS regions, including the hypothalamus [19]. These proliferating cells are scattered in a large hypothalamic parenchymal area around the third ventricle, and many of them express neuronal markers. Similarly, the icv infusion of CNTF in adult mice is exerting a strong anorexigenic effect leading to long-lasting weight loss that is dependent upon the de novo generation of neuronal cells [18]. In fact, CNTF-treated adult mice showed accumulation of BrdU-positive cells in the hypothalamus, especially in the ventromedial and arcuate nuclei, two areas involved in the control of feeding. The present data are therefore consistent with these studies [18], [19] and support the hypothesis that putative neural precursor cells/stem cells residing in the adult hypothalamic parenchyma are normally in a quiescent state, but capable of responding to stimulation by opportune exogenous factors such as BDNF and CNTF.

We therefore reasoned that CNTF or BDNF should have a similar proliferative effect *in vitro*. However, despite many attempts and the fact that the receptors for these molecules are expressed in our system, we never succeeded in generating primary NS when the two growth factors EGF or FGF-2 were substituted by either CNTF, BDNF or a combination of the two. It therefore appears that the putative CNTF/BDNF-responsive cells residing within the hypothalamic parenchyma are either not present or not capable of responding to stimulation in the primary neural cell cultures. One possible explanation would be that even though CNTF/BDNF is able to induce neurogenesis in vivo, it may not be sufficient to support all the physiological processes required for the in vitro generation of NS and that other factors may interact with CNTF/BDNF to promote proliferation. Another possibility could be that EGF and FGF-2 responsive cells are already committed for proliferation in vivo [29], [30], and that they maintain this characteristic in vitro, resulting in a prompt response to stimulation. Other cell types may be less robust, or loose their commitment toward proliferation once away from their natural microenvironment.

We were also able to demonstrate the presence of GnRH-expressing cells among NS derived from fetal (E18) cultures when they were kept in differentiating conditions. This is somewhat surprising considering the embryonic origin of GnRH neurons, which are thought to derive from migratory cells originating from the olfactory placode [31]. However, more recent data suggest that GnRH-expressing cells may have multiple embryonic origins, a hypothesis that would be consistent with the present results [32]. Moreover, data derived from transgenic mice expressing the LacZ reporter gene under the control of the GnRH promoter indicate that GnRH-expressing cells can be transiently detected in the forebrain during embryogenesis [33]. Finally, GnRH-immunoreactive cells derived from in vitro expanded progenitor cells were also reported from either adult hypothalamic or hippocampal cells [14]. For the latter studies, the authors used a density gradient centrifugation technique to isolate progenitor cells from either the hypothalamus or the hippocampus, and they were able to detect many GnRH-immunoreactive cells in the cultures originated from both CNS regions. They suggest that when put in vitro, progenitor cells are freed of the constraints of their embryonic origin and can therefore generate different subtypes of neurons than they would do in vivo. This hypothesis could possibly explain the present results as well, but our study differs from the previously published experiments [14] in the following ways: the techniques used for the isolation of precursor cells were different, and so were the conditions of differentiation applied. Another important difference between our study and that of Markakis et al. [14] is that we could detect GnRH-immunoreactive cells only in fetal but not in adult hypothalamic cultures. Markakis et al. used 7 week-old Fisher rats to derive progenitor cells and we used Wistar rats, between 8 to 10 weeks of age, for the adult hypothalamic cultures. Therefore, we cannot rule out the possibility that either a difference in age or/and in the genetic background could explain these discrepancies. Alternatively, GnRH progenitors may reside in the adult SVZ, which was not isolated in our culture system. Nevertheless, our data and these previous studies [33], [14] together suggest that GnRH-expressing neurons could arise from central nervous system progenitor cells, but further work will be necessary to assess the functional significance of this observation.

In conclusion, we report here that using a simple and robust culture protocol, both the fetal and adult hypothalami are a rich source of NS that can give rise to cells of all three lineages of the central nervous system. This capacity of the adult hypothalamic parenchyma to generate NS does not depend upon the presence of the ependymal layer of the third ventricle, and the neuronal cells thus obtained still express specific hypothalamic molecular markers. Finally, we were able to detect GnRH-expressing neurons among cells derived from these fetal NS, and we therefore suggest that this culture system represents a useful model to study the molecular mechanisms of GnRH neuronal cell differentiation, a central process for the neuroendocrine control of reproduction in mammals.

## Materials and Methods

### Hypothalamic dissection and NS cell cultures

Adult (8 to 10 weeks old) and fetal (E18) Wistar rats were used for this study (Charles River, Les Oncins, France). Animals were killed by decapitation and hypothalami were dissected out of brains and immediately placed into sterile trituration solution (0.1% Glucose, 1% Penicillin-Streptomycin in PBS). Under a tissue culture hood, the hypothalamic tissue was washed 3 times in sterile trituration solution to eliminate residual blood. Mechanical dissociation of hypothalamic tissue was done by gentle pipetting up and down with a sterile glass Pasteur pipette. This procedure was repeated 3 times, each time recovering the dissociated tissue away from tissue debris. Pooled dissociated tissue fractions were then centrifuged (5 minutes at 700–800 rpm) and resulting cell pellets suspended in 2–4 mL (volume depending on the amount of starting tissue) of culture media (DMEM/F12 supplemented with 0.5% B-27 and 20 ng/mL of either FGF-2 or EGF growth factors). Trypan Blue staining was used to count viable cells and cells were seeded into either 6-well or 12-well plates at a density of 10–20,000 cells/mL. Fresh media to compensate for evaporation, together with growth factors, was added every 4 days. NS were passaged by mechanically dissociating the cell clusters into single cells with the help of yellow pipette tips and reseeding at a density of 10,000 cells/mL into new vessel plates. For long term storage proliferating NS were kept in liquid nitrogen, in their standard media (DMEM:F12, 0.5% B-27) supplemented with 1% BSA and 10% DMSO.

### Removal of third ventricle and surrounding tissue

In order to remove third ventricle tissue, adult rats were anesthetized with chloral hydrate (400 mg/Kg bw i.p.) and stereotactically injected with either a solution of methylene blue dye (10 μL) or DiI (10 μL of 1 mM, Vibrant DiI, Invitrogene) into the right lateral ventricle (coordinates: 1 mm posterior,2 mm lateralfrom bregma and 4.5 mm dorsoventral below skull). Following icv injection, animals were sacrificed by decapitation, and the brain was removed. The time elapsed between icv infusion and sacrifice was 10–15 minutes. Hypothalami were dissected out of brains and the blue/red-stained ventricular tissue gently removed by using a scalpel, then the remaining hypothalamic parenchyma was processed for cell culture as described above.

### RT-PCR

Total RNA was extracted from either pooled neurospheres or differentiating cultures by using the TriPure® reagent (Roche). Extracted RNA was quantitated by spectrophotometry and quality evaluated by running samples on denaturing agarose gels. cDNA was prepared from 100–250 ng of total RNA using SuperscriptII (Invitrogene) enzyme and random primers, according to manufacturer's instructions. The primer pairs used for the PCR reactions are shown on Table 1.

**Table 1:** Primers used for the PCR analysis.

Gene	sense	antisense
*NSE*	5′-AACCTGATGCTGGAGTTGGATG-3′	5′-CCTTGTCAATGGCTTCCTTCAC-3′
*GFAP*	5′-AGCTCAATGACCGCTTTGCTAGC-3′	5′-GACTCAACCTTCCTCTCCAGATCCA-3′
*Nestin*	5′-AGTGTCGCTTAGAGGTGCAACAGC-3′	5′-TTCAGGAAGGCTGTCACAGGAGTC-3′
*ß-2m*	5′-GAGCCCAAAACCGTCACCT-3′	5′-GAAGATGGTGTGCTCATTGC-3′
*EgfR*	5′-CATGCGGAACTTACAGGAAA-3′	5′-TCACCAATGCCTATGCCA-3′
*FgfR1*	5′-CATCACTCTGCATGGTTGACC-3′	5′-TCAGGTGCCATCCACTTCA-3′
*CntfR*	5′-CGATCCTCCAGAAAATGTGG-3′	5′-TCAGGAGATTGTTGGCTGTG-3′
*TrkB*	5′-CCTCTATGAAGACTGGACCACG-3′	5′-ATTGTCGCTGGCGTCCTT-3′
*Gp130*	5′-TCAACTTGTGGAACCATGTGG-3′	5′-TCCAACTGACACAGCATGTTC-3′
*LIFR*	5′-GCCTCCTGGACTGGATAAAGGTTC-3′	5′-ACAAACATGCTCTTCTCTGGGCC-3′
*Otp*	5′-ATGCTGTCTCACGCCGACCT-3′	5′-ACCAGACCTGCACTCGCGAT-3′
*Arnt2*	5′-CGTTGTGTGGCTACTGTTGG-3′	5′-CTTAGCTCGGAATCGGAACA-3′
*Brn2*	5′-CTAGGCCAGCCGGACATCAA-3′	5′-TGCATGATGGGGCTCATCG-3′
*Ttf1*	5′-AAATTTGGGGGTCTTTCTGG-3′	5′-AGAGTGCATCCACAGGGAAG-3′
*Pit1*	5′-CGTGAATCGGCCCTTTGATACA-3′	5′-ACTTTTCCGCCTGAGTTCCTGC-3′

### Immunocytochemistry

To induce differentiation, proliferating NS were mechanically dissociated into single cells and seeded on 12-well plates containing glass coverslips treated with poly-L-Ornithine and cultured in DMEM:F12, 0.5% B27, 1% FBS, without growth factors. After 7 days in culture, cells were fixed in 4% Paraformaldehyde with 20 min incubation and then kept refrigerated in PBS till use. All primary antibodies used in the study were incubated overnight at 4°C. All secondary antibodies used in this study were incubated for 1 h at room temperature. Counterstaining was performed by incubating coverslips with DAPI solution (Molecular Probes, Eugene, OR) for 5 minutes. All coverslips were mounted on glass slides with the Mowiol solution (Calbiochem, Darmstadt, Germany).

Primary antibodies used in this study were the following: mouse IgG monoclonal anti-ß-tubuline III (Sigma, Buchs, Switzerland) used at 1∶1000 dilution, rabbit anti-GFAP antiserum (Dako, HighWycombe, UK) used at 1∶2000 dilution, rabbit anti-GnRH antibody (Chemicon, Basel, Switzerland) used at 1∶5000 dilution, rabbit polyclonal anti-GnRH antiserum [25] used at a 1∶200 dilution and mouse IgM monoclonal anti-O4 (Chemicon, Basel, Switzerland) used at 1∶500,. Secondary antibodies ( Jackson Immuno-Research, West grove, PA) used in this study were the following: FITC-conjugated goat antibody to rabbit IgG used at 1∶100 dilution, Rhodamine-conjugated goat antibody to mouse IgG used at 1∶50 dilution, Rhodamine-conjugated goat antibody to mouse IgM used at 1∶200 dilution, Rhodamine-conjugated goat antibody to rabbit IgG used at 1∶100 dilution..
